# Epicardial and visceral adipose tissue in relation to subclinical atherosclerosis in a Chinese population

**DOI:** 10.1371/journal.pone.0196328

**Published:** 2018-04-25

**Authors:** Nang Ei Ei Khaing, Tai E. Shyong, Jeannette Lee, Cinnie Yentia Soekojo, Alvin Ng, Rob M. Van Dam

**Affiliations:** 1 Saw Swee Hock School of Public Health, National University of Singapore, Singapore, Republic of Singapore; 2 Department of Medicine, National University Health System, Singapore, Republic of Singapore; 3 Mount Elizabeth Medical Centre, Singapore, Republic of Singapore; 4 Department of Endocrinology, Singapore General Hospital, Singapore, Republic of Singapore; 5 Department of Nutrition, Harvard T.H. Chan School of Public Health, Boston, Massachusetts, United States of America; Beijing Key Laboratory of Diabetes Prevention and Research, CHINA

## Abstract

**Background:**

Body fatness is associated with risk of coronary heart disease and it has been postulated that epicardial adipose tissue (EAT) may have a particularly detrimental effect because of its localized toxic effects. We therefore aimed to examine the association between EAT and coronary artery calcification and compared this with associations for visceral adipose tissue (VAT) and other regional fat depots.

**Methods:**

We conducted a cross-sectional study of 487 Chinese participants aged 50 years old and above, living in Singapore. Participants, free from known diabetes mellitus and coronary heart diseases, completed interviews, a health screening to evaluate obesity and cardiovascular disease risk factors, and computed tomography scans of the abdomen and coronary arteries. Associations between regional fat depots and subclinical atherosclerosis defined as CAC> = 100 were determined by multiple logistic regression analysis.

**Results:**

Epicardial adipose tissue (EAT) was highly correlated with visceral adipose tissue (VAT) (Pearson r = 0.72) and trunk fat mass (r = 0.66). The age and sex-adjusted odd ratio (OR) (in 1-SD increase) of subclinical atherosclerosis was 1.28 (1.01–1.61) for EAT and 1.40 (1.04–1.88) for VAT. These associations were weaker and non-significant after adjusting for markers of dyslipidemia and hyperglycemia. Total body fat, subcutaneous abdominal fat, and leg, arm and trunk fat mass were not significantly associated with atherosclerosis.

**Conclusion:**

VAT and EAT showed similar associations with coronary artery calcification and the associations could be mediated by traditional risk factors in this ethnic Chinese population.

## Introduction

Obesity is an independent risk factor for cardiovascular diseases and the distribution of body fat is an important predictor of cardiovascular disease risk [1]. Certain fat depot such as visceral adipose tissue (VAT) or epicardial adipose tissue (EAT) may impose higher risk than other fat depots [1]. VAT and EAT have the same embryological origin and the size of both fat depots has been shown to be associated with coronary artery calcification [2, 3] and coronary heart disease (CHD) [4, 5]. Some authors suggested that EAT has a stronger association with CHD than other adiposity indexes including VAT due to its localized toxic effect, [6] whereas other authors suggested that VAT imposes a higher risk due to its systematic effects [7].

Most of these studies have been carried out in western population and it has been reported that there is an ethnic influence on the association of regional fat and cardiovascular disease (CVD) risk factors [8, 9]. Few studies have assessed the association between EAT, VAT, regional fat and coronary artery calcification in Asian populations [6, 10]. These studies were conducted in specific populations such as patients with diabetes or those referred to cardiovascular departments and results on the effect of epicardial fat on coronary artery calcification compared with other regional fat depots may thus not be generalizable to the general population. We therefore aimed to determine the association between epicardial fat and coronary artery calcification and subsequently to compare the relation of EAT and other regional fat depots on coronary artery calcification in a population-based study in ethnic Chinese adults.

## Materials and methods

### Study design and study population

This analysis was based on cross-sectional data of 487 participants of a follow-up round of the Singapore prospective study [11]. The Singapore Prospective Study Program (2004–2007) is a population-based cross-sectional study of CVD in a multi-ethnic population in Singapore. A total of 10,445 participants from four previous cross-sectional studies: Thyroid and Heart Study 1982–1984, National Health Survey 1992, National University of Singapore Heart Study 1993–1995 and National Health Survey 1998, were invited and 7744 (76.8% response rate) participants were interviewed, and 5164 (66.7%) attended the health examination. Details of the study methods have been described elsewhere [11].

These participants were invited for a follow-up study (2011–2016). From the participants of the follow-up study, the first 808 participants who met the eligibility criteria (aged above 50 years old without a history of heart failure, heart attack, stroke, kidney failure, or cancer, and not treated with high-dose steroids) were invited to participate in the current study with more detailed physiological measurements. Participants were invited to undergo the interview and health examination including a computed tomography (CT) scan on a different day. Of the 808 eligible individuals, 2 refused and 1 was unable to undergo the CT scan due to asthma and a high heart rate. Of the 805 who underwent coronary CT scans, 788 completed the questionnaire and 801 provided blood samples. A total of 784 participants completed the questionnaire, provided blood samples and underwent coronary CT scans. From these 784 participants, we excluded participants with non-Chinese ethnicities due to small numbers (N = 45), from the same household (N = 36), with extremely high triglyceride levels (>12.4 mmol/L) (N = 1), with known or newly diagnosed diabetes, (N = 105), coronary artery disease (N = 6), or cancer (N = 1) and those on lipid medication (N = 217). For the VAT and SAT analysis, we further excluded participants with missing abdominal CT scans (N = 91). Participants could be excluded for one or more reasons. There was a total of 487 for the analysis of EAT and body composition and 396 participants for analysis of VAT and SAT.

### Ethics statements

Ethics approval was obtained from 2 institutional review boards (the National University of Singapore and the Singapore General Hospital). Written informed consent was obtained from each participant before the study was conducted.

### Assessment of coronary artery calcium

A non-contrast CT scan for coronary calcium scoring was performed using prospective gating typically at 45–60% of R–R interval (120 kVp, 1.4 s per cycle). Coronary artery calcium (CAC) was assessed by a 64-slicer multi-detector GE Light Speed volume CT (GE Medical Systems, Milwaukee, Wisconsin).

A traditional Agatston score was calculated for each scan, defining calcium as 2 adjacent pixels > = 130 Hounsfield units (HU). The lesion score was calculated by multiplying the lesion area by a weighting factor (1 = 130 to 200 HU, 2 = 201 to 300 HU, 3 = 301 to 400 HU, and 4 = ≥401 HU) as described by Agatston et al. (1990). A total calcium score was determined by summing individual lesion scores from the standard 4 cardiac vessels (left main, left anterior descending, left circumflex, and right coronary artery) and may include the scores from other vessels (Eg, posterior descending artery) if they become prominent. The total CAC score was calculated according to the Agatston-Janowitz 130 score [12].

Subclinical atherosclerosis was defined as CAC > = 100 [13].

### Assessment of adipose tissues

Epicardial and abdominal adipose tissue were measured from CT scans by sliceOmatic version 5.0 (Tomovision, Magog, Canada) using a semi-automatic segmentation technique. Epicardial fat was defined as the adipose tissue located within the surface of the heart and the visceral layer of the pericardium (visceral epicardium) [14]. VAT was defined as adipose tissue inside the abdominal muscular wall and SAT as adipose tissue outside the abdominal muscular wall [15]. The readers manually traced the region of interest and within the region of interest, adipose tissue was defined as the pixels between -190 to -30 Hounsfield units [16].

We measured EAT at the level of the left main coronary artery (LMCA) as it is shown to be most strongly correlated with epicardial fat volume [17]. One reader made all the measurements. The reproducibility was assessed from randomly selected 15 CT scans and the mean % intra-observer coefficient of variation (CV) was 4.86. We also assessed the inter-observer CV from randomly selected 29 CT scans to ensure reproducibility and the mean %CV was 5.07.

For abdominal adipose tissue, the measurements were made at inter-vertebral space L2/L3 and L4/L5 level. A single observer identified inter-vertebral space L2/L3 and L4/L5 levels by efilm work station version 4.0 (Hartland, USA). Each image was analyzed by two independent readers and the mean of the two readings was taken as final measurement. We calculated inter-observer CV from all 581 scans and average inter-observer CV for SAT at L2/L3 and L4/L5 were 2.34% and 1.80% and for VAT at L2/L3 and L4/L5 were 3.22% and 3.13% respectively. In this study, we presented SAT and VAT at L2/L3 because VAT at L2/L3 was previously shown to be the best level to estimate the total VAT volume and better correlated with cardiovascular risk factors than VAT at L4/L5 in Asian Chinese [18].

### Assessment of body composition

Arms, legs, trunk and total fat mass (FM), and total body fat percent (%BF) were assessed by dual-energy x-ray absorptiometry (DXA) imaging (Discovery Wi; Hologic, Bedford, MA and software Hologic Apex 3.01) using a medium speed total body acquisition mode.

### Assessment of risk factors

Central aortic blood pressure was measured by A-PULSE CASPro and the brachial blood pressure was measured by digital blood pressure monitor (Healthstats or Dinamap Pro100V2) and mean value of the two readings was calculated. If the difference between two readings was greater than 10mmHg for systolic blood pressure (SBP) and/or greater than 5mmHg for diastolic blood pressure (DBP), a third reading was taken and mean value of the second and the third readings was calculated. As the results were similar, we presented only the results for central aortic blood pressure. Hypertension was defined as a systolic blood pressure > 140 mmHg or diastolic blood pressure > 90 mmHg or a history of hypertension, or currently taking anti-hypertensive medications.

For blood tests, participants were instructed to fast overnight for at least 10 hours. Venous blood was drawn and collected in plain, EDTA, and fluoride oxalate tubes. Blood tubes were left to stand for 30 minutes at room temperature before storing them at 4°C. The samples were sent to the Singapore General Hospital laboratory for analysis where serum was tested for creatinine, glucose, total cholesterol, high density lipoprotein cholesterol (HDL-C), low density lipoprotein cholesterol (LDL-C) and triglycerides (TG) using a chemistry analyzer (Beckman Coulter Unicel DxC 800). Glycated haemoglobin A1C (HbA1c) levels were measured using immunoassay (Roche Cobas c501).

High Cholesterol was taken as present if the participant answered yes to the question of “Have you ever been told by a physician (Western-trained) you have high cholesterol?”. History of smoking was assessed by asking whether they ever smoked cigarettes in their lifetime and if they currently smoked cigarettes. Current alcohol drinking was assessed by asking the participants’ alcohol consumption in the past 30 days.

### Statistical analysis

All statistical analyses were performed using Stata 12 for Windows (Stata Corporation, College station, Texas, USA). In Table 1, for normally distributed variables, means and standard deviations were presented and for skewed variables, medians and inter-quartile ranges were presented. Pearson’s correlation was used to test the correlation between regional measures of body composition in Table 2 and the association between body composition and CVD risk factors, adjusting for age and sex in Table 3. In Table 4, association between regional fats and subclinical atherosclerosis were examined by logistic regression analysis, adjusting for age and sex. In Table 5, multiple logistic regression analysis was used to assess the associations between VAT, EAT and subclinical atherosclerosis respectively. To understand the possible mediating role of CVD risk factors in these associations, we added risk factors in each model; the basic model was adjusted for age and sex with additional adjustment of smoking and hypertension in model 1, LDL-C and triglycerides/HDL-C in model 2and HbA1c in model 3. All statistical tests were two-sided with a level of significance defined as a p-value < 0.05.

**Table 1:** Characteristics of the study population.

Age, years, mean (SD)	58.23 (6.39)
Sex, N (%)	
Male	236 (48.46)
Female	251 (51.54)
Smoking, N (%)	
Never smoker	355 (72.90)
Ex-smoker	41 (8.42)
Current smoker	91 (18.69)
Hypertension (history or newly diagnosed), N (%)	231 (47.43)
History of high cholesterol, N (%)	93 (21.04)
Current alcohol drinking, N (%)	94 (19.30)
Presence of CAC (CAC>0), N (%)	246 (50.51)
Subclinical atherosclerosis (CAC > = 100), N (%)	77 (15.81)
Central aortic systolic blood pressure (mmHg), mean (SD)	133.77 (19.76)
Central aortic diastolic blood pressure (mmHg), mean (SD)	82.47 (10.68)
Systolic blood pressure (mmHg), mean (SD)	135.29 (20.33)
Diastolic blood pressure (mmHg), mean (SD)	83.11 (10.73)
Fasting plasma glucose (mmol/L), mean (1SD range)^a^	4.93 (4.42–5.50)
HBA1C (%), mean (SD)	5.64 (0.40)
Total cholesterol (mmol/L), mean (SD)	5.69 (0.93)
HDL- cholesterol (mmol/L), mean (SD range) ^a^	1.45 (1.08–1.93)
LDL- cholesterol (mmol/L), mean (SD)	3.71 (0.85)
Triglycerides (mmol/L), mean (1SD range) ^a^	1.10 (0.67–1.81)
Triglycerides/HDL, mean (1SD range) ^a^	0.76 (0.38–1.53)
EAT (cm^2^), mean (SD)	0.11 (0.06)
Arm fat mass (kg), mean (SD)	1.21 (0.42)
Leg fat mass (kg), mean (SD)	3.27 (1.02)
Trunk Fat mass (kg), mean (SD)	10.32 (3.37)
Total fat mass (kg), mean (SD)	20.35 (5.74)
Total body fat %, mean (SD)	33.84 (7.35)
VAT (cm^2^), mean (SD)	106.47 (63.92)
SAT (cm^2^), mean (SD)	111.59 (49.74)

^a^ log transformed and back-transformed

CAC = coronary artery calcium; HBA1C = Glycated haemoglobin A1C, HDL-C = high density lipoprotein cholesterol, LDL-C = low density lipoprotein cholesterol, EAT = epicardial adipose tissue measured at Left main coronary artery, VAT = visceral adipose tissue and SAT = subcutaneous adipose tissue measured at L2/L3

The sample size is 487 except for VAT/SAT is 396.

**Table 2:** Pearson's correlation between regional fat depots.

N = 396	EAT at LMCA	Arm fat mass	Leg fat mass	Trunk Fat mass	Total fat mass	Total body fat %	VAT	SAT
EAT	1.00							
Arm fat mass	0.49	1.00						
	(<0.0001)							
Leg fat mass	0.28	0.78	1.00					
	(<0.0001)	(<0.0001)						
Trunk fat mass	0.66	0.83	0.66	1.00				
	(<0.0001)	(<0.0001)	(<0.0001)	(<0.0001)				
Total fat mass	0.57	0.91	0.86	0.95	1.00			
	(<0.0001)	(<0.0001)	(<0.0001)	(<0.0001)				
Total body fat %	0.22	0.74	0.80	0.60	0.73	1.00		
	(<0.0001)	(<0.0001)	(<0.0001)	(<0.0001)	(<0.0001)			
VAT	0.72	0.44	0.20	0.71	0.57	0.08	1.00	
	(<0.0001)	(<0.0001)	(0.0001)	(<0.0001)	(<0.0001)	(0.12)		
SAT	0.44	0.84	0.72	0.83	0.87	0.75	0.33	1.00
	(<0.0001)	(<0.0001)	(<0.0001)	(<0.0001)	(<0.0001)	(<0.0001)	(<0.0001)	

Data is presented in Pearson’s correlation and p value in the bracket.

EAT = epicardial adipose tissue measured at left main coronary artery, VAT = visceral adipose tissue and

SAT = subcutaneous adipose tissue measured at L2/L3

**Table 3:** Adjusted Pearson's correlation between regional body fat depots and risk factors.

	Central aortic systolic blood pressure	Central aortic diastolic blood pressure	Fasting plasma glucose ^a^	HBA1C (%)	Total cholesterol	HDL-cholesterol ^a^	Triglycerides ^a^	Triglycerides/HDL ^a^	LDL-cholesterol
Arm fat mass (kg)	0.17 (0.0002)	0.24 (<0.0001)	0.27 (<0.0001)	0.18 (0.0001)	-0.08 (0.07)	-0.33 (<0.0001)	0.24 (<0.0001)	0.31 (<0.0001)	0.05 (0.24)
Leg fat mass (kg)	0.08 (0.08)	0.13 (0.004)	0.18 (<0.0001)	0.10 (0.03)	-0.05 (0.32)	-0.16 (0.0003)	0.08 (0.07)	0.13 (0.006)	0.06 (0.21)
Trunk fat mass (kg)	0.19 (<0.0001)	0.30 (<0.0001)	0.33 (<0.0001)	0.22 (<0.0001)	-0.02 (0.73)	-0.36 (<0.0001)	0.31 (<0.0001)	0.37 (<0.0001)	0.11 (0.01)
EAT (cm^2^)	0.17 (0.0001)	0.21 (<0.0001)	0.28 (<0.0001)	0.21 (<0.0001)	0.05 (0.26)	-0.27 (<0.0001)	0.29 (<0.0001)	0.32 (<0.0001)	0.12 (0.009)
Total fat mass (kg)	0.16 (0.0003)	0.26 (<0.0001)	0.30 (<0.0001)	0.19 (<0.0001)	-0.04 (0.43)	-0.32 (<0.0001)	0.25 (<0.0001)	0.31 (<0.0001)	0.09 (0.04)
Total body fat %	0.14 (0.002)	0.23 (<0.0001)	0.27 (<0.0001)	0.18 (0.0001)	0.01 (0.90)	-0.25 (<0.0001)	0.21 (<0.0001)	0.25 (<0.0001)	0.13 (0.005)
VAT (cm^2^)	0.18 (0.0003)	0.29 (<0.0001)	0.32 (<0.0001)	0.26 (<0.0001)	0.01 (0.78)	-0.37 (<0.0001)	0.39 (<0.0001)	0.43 (<0.0001)	0.10 (0.05)
SAT (cm^2^)	0.14 (0.004)	0.24 (<0.0001)	0.22 (<0.0001)	0.14 (0.004)	-0.05 (0.37)	-0.20 (<0.0001)	0.17 (0.0006)	0.20 (0.0001)	0.06 (0.20)

^a^ log transformed

Data is presented in Pearson’s correlation, adjusting for age and sex and p value in the bracket.

HBA1C = Glycated haemoglobin A1C, HDL-C = high density lipoprotein cholesterol, LDL-C = low density lipoprotein cholesterol, EAT = epicardial adipose tissue measured at left main coronary artery, VAT = visceral adipose tissue and SAT = subcutaneous adipose tissue measured at L2/L3

The sample size is 487 except for VAT/SAT is 396.

**Table 4:** Association between regional body fat depots and subclinical atherosclerosis.

	Subclinical atherosclerosis (CAC> = 100)		
	OR	95% CI	
EAT (in 1SD)	1.28*	1.01	1.61
VAT (in 1SD)	1.40*	1.04	1.88
SAT (in 1SD)	1.13	0.82	1.56
Arm fat mass (in 1SD)	1.14	0.87	1.49
Leg fat mass (in 1SD)	1.30	0.99	1.72
Trunk fat mass (in 1SD)	1.22	0.95	1.56
Total fat mass (kg)	1.25	0.97	1.61
Total body fat % (in 1SD)	1.07	0.74	1.55

Adjusted for age and sex

**Table 5:** Association of EAT, VAT and subclinical atherosclerosis.

	Subclinical atherosclerosis								
	Model 1			Model 2			Model 3		
	OR	95% CI		OR	95% CI		OR	95% CI	
**VAT**									
VAT (in 1SD)	1.43*	1.05	1.94	1.20	0.86	1.69	1.12	0.79	1.59
Age	1.09***	1.05	1.14	1.11***	1.06	1.16	1.10***	1.05	1.16
Female	0.58	0.28	1.21	0.62	0.29	1.34	0.56	0.26	1.23
Current smoker	1.34	0.63	2.85	1.22	0.56	2.63	1.26	0.57	2.76
Ex-smoker	0.65	0.21	1.96	0.74	0.24	2.26	0.75	0.24	2.33
Hypertension	1.06	0.58	1.96	0.99	0.53	1.84	0.97	0.52	1.81
LDL				1.35	0.94	1.94	1.29	0.89	1.87
Triglycerides/HDL^a^				1.91*	1.17	3.14	1.73*	1.05	2.86
HbA1c (%)									2.82*	1.26	6.31
**EAT**									
EAT (in 1SD)	1.30*	1.03	1.65	1.21	0.95	1.56	1.16	0.90	1.50
Age	1.08***	1.04	1.12	1.09***	1.05	1.13	1.09***	1.05	1.13
Female	0.39*	0.22	0.71	0.44*	0.24	0.81	0.41*	0.22	0.75
Current smoker	1.15	0.60	2.20	1.12	0.58	2.15	1.14	0.58	2.21
Ex-smoker	0.74	0.30	1.84	0.80	0.32	1.99	0.81	0.32	2.03
Hypertension	0.91	0.54	1.54	0.86	0.50	1.46	0.82	0.48	1.40
LDL				1.16	0.85	1.58	1.10	0.80	1.51
Triglycerides/HDL ^a^				1.42	0.95	2.12	1.29	0.86	1.94
HbA1c (%)							2.29*	1.17	4.51

^a^ log transformed

*p value<0.05

*** p value<0.0001

Model 1: Adjusted for age, sex, smoking and hypertension

Model 2: Adjusted for variables in Model 1 plus LDL and Triglycerides/HDL

Model 3: Adjusted for variables in Model 2 plus HbA1c

EAT = Epicardial adipose tissue measured at left main coronary artery, VAT = visceral adipose tissue and SAT = subcutaneous adipose tissue measured at L2/L3, CAC = coronary artery calcium, HBA1C = Glycated haemoglobin A1C, HDL-C = high density lipoprotein cholesterol, LDL-C = low density lipoprotein cholesterol

SD = standard deviation, OR = odds ratio, CI = confidence interval

The sample size for VAT is 396 and for EAT is 487

## Results

Table 1 shows the characteristic of the study population. The mean age of the population was 58 years (SD = 6) and 52% were female. Of the participants, 51% had presence of CAC and 16% had subclinical atherosclerosis.

Table 2 shows the correlation between the sizes of the regional fat depots. EAT was highly correlated with VAT (r = 0.72) and trunk fat mass (r = 0.66), moderately correlated with total fat mass (r = 0.57), arm fat mass (r = 0.49) and SAT (r = 0.44) and only weakly correlated with leg fat mass (r = 0.28) and total body fat percent (r = 0.22)

Table 3 shows the correlation between different measures of body fat and CVD risk factors, adjusting for age and sex. Most measures of adiposity were associated with blood pressure and glucose, Hba1c, triglyceride and HDL-cholesterol concentrations. Additionally, EAT, VAT, trunk fat, total fat mass and percentage body fat were significantly associated with higher LDL-cholesterol concentrations.

Table 4 shows the adjusted association between body composition and subclinical atherosclerosis. EAT and VAT were significantly associated with subclinical atherosclerosis after adjusted for age and sex. No significant association was observed between other regional fats and subclinical atherosclerosis.

Table 5 presents associations of VAT and EAT with subclinical atherosclerosis. Both VAT (OR: 1.43, 95% CI: 1.05–1.94) and EAT (OR: 1.30, 95% CI: 1.03–1.65) remained associated with subclinical atherosclerosis after adjusting for age, sex, smoking and hypertension. However, these associations became substantially weaker and non-significant after additional adjustment for dyslipidemia and hyperglycemia; the odds ratio of subclinical atherosclerosis became 1.12 for VAT (95% CI: 0.79–1.59) and 1.16 for EAT (95% CI: 0.90–1.50). We conducted additional analysis by excluding participants with high HbA1C levels (> = 6.5%) or hyperlipidemia and there was no substantial change in the results (data not shown).

## Discussion

In our study of ethnic Chinese adults, EAT and VAT were associated with subclinical atherosclerosis after adjusting for age, sex, smoking and hypertension. Other body fat depots were not significantly associated with subclinical atherosclerosis. Adjustment for markers for dyslipidemia and hyperglycemia substantially weakened these associations suggesting that the effect of EAT and VAT on atherosclerosis could be mostly mediated by these cardiovascular risk factors.

VAT was more strongly correlated with CVD risk factors than EAT. In the Framingham Heart Study, VAT was also more strongly correlated with metabolic risk factors than other fat depots [19]. We observed that both EAT and VAT were associated with subclinical atherosclerosis. This is consistent with the findings from previous studies. Several studies have been conducted on the association between EAT and subclinical atherosclerosis [2, 19, 20]. In a U.S. study done in patients who underwent a CT angiogram and echocardiography for the diagnosis of CAD, EAT was significantly associated with coronary calcification and coronary atheromatous plaque [20]. In a study in Japanese adults, VAT but not SAT was associated with a higher risk of atherosclerosis measured by carotid intima media thickness [21]. Similarly, in the Multicultural Community Health Assessment Trial, only VAT but not SAT and total fat area was positively correlated with intima media thickness [22]. However, in a study done in 174 patients in Japan, EAT but not VAT was found to be associated with CAC [20]. It may be due to the difference in the cutoff point for CAC. Our study used CAC>100 while the aforementioned study used CAC>0. When we used CAC>0, we observed similar finding (data not shown).

These associations of EAT and VAT with subclinical atherosclerosis were substantially weakened after adjustment for markers of dyslipidemia and hyperglycemia. This finding suggests that effects of EAT and VAT on atherosclerosis are partly mediated by these metabolic risk factors, but does not exclude the possibility that EAT and VAT may also have independent effects on CHD over and above traditional cardiovascular risk factors. For example, in the Heinz Nixdorf Recall Study with 5-year follow up, EAT volume was associated with a 20% progression of CAC after adjusting for traditional cardiovascular risk factors and this association was statistically significant [23]. Moreover, EAT remained associated with fatal and non-fatal cardiovascular events after adjusted for traditional cardiovascular risk factors and CAC in this study [24]. VAT also remained significantly associated with intima media thickness in the Multicultural Community Health Assessment Trial after accounting for cardiovascular biomarkers [22]. In combination, the results of these studies suggest that the effect of EAT and VAT on atherosclerosis may only be partially mediated by traditional cardiovascular biomarkers.

To our knowledge, this is the first study which simultaneously assessed EAT and regional fat distribution with CAC in an Asian population. We comprehensively assessed regional fat depots using detailed measurements such as CT and DXA scans. However, our study also had limitations that should be considered. First, EAT and VAT measurements can still be affected by measurement error, which may have weakened observed associations. In particular, we did not directly measure total epicardial fat volume. However, EAT at LMCA has been shown to be highly correlated with total epicardial fat volume [17]. Second, our cross-sectional study design does not allow us to make definite conclusions about the causal direction of observed associations. Third, our sample size was modest this may have limited our statistical power to detect significant associations. Finally, it should be noted that CAC is a surrogate marker for CHD, but does not capture plaque stability.

In a meta-analysis, the relative risk of CHD was 2.6 (95% CI, 1.7–4.0) for a CAC score < 100, 8.8 (95%CI, 4.1–19.0) for a score of 101 to 400, and 17.0 (95% CI, 6.9–40.0) for a score >400 [25]. In our study of ethnic Chinese adults, both EAT and VAT were associated with measures of CAC suggesting that they may have a similar impact on atherosclerosis that is larger than that of other body fat depots. Furthermore, the effects of EAT and VAT on atherosclerosis may be partly mediated by traditional cardiovascular risk factors particularly dyslipidemia and hyperglycemia.
